# Focus on Lactate and Lactylation Modification: The Potential Role in Ophthalmic Disease Treatment

**DOI:** 10.3390/ijms27052516

**Published:** 2026-03-09

**Authors:** Mengyu Zong, Yu Qiu, Changyong Li

**Affiliations:** Institute of Rehabilitation Medicine, Qilu Medical University, Zibo 255200, China

**Keywords:** lactylation, ocular disease, post-translational modification, glycolysis

## Abstract

Lysine lactylation represents a novel post-translational modification (PTM) involved in cellular functions including glycolysis and macrophage polarisation. It differs in form and mechanism from other PTMs such as acetylation, methylation, phosphorylation, ubiquitination, and SUMOylation. As a recently discovered modification, lactylation has been implicated in the progression of multiple diseases. Recent studies further indicate lactylation’s association with multiple ocular pathologies. This review systematically summarises and discusses lactylation’s involvement in prevalent eye diseases, including myopia, retinopathy, ocular melanoma, uveitis, and macular degeneration. We further collate emerging data suggesting lactylation signalling pathways may represent potential therapeutic targets for ocular pathologies. This review aims to provide a comprehensive overview for holistic intervention strategies and multidimensional assessment across various ocular conditions, while offering valuable insights for future research and development from a lactylation perspective.

## 1. Background

PTMs play a role in maintaining homeostasis. PTMs were first discovered in 1906, when the phenomenon of protein phosphorylation in ovalbumin was revealed. With extensive research, new observations concerning PTMs have rapidly emerged. PTMs regulate DNA-dependent processes including transcription, replication, and DNA repair. These modifications exert their effects by altering nucleosome-DNA interactions or recruiting non-histone proteins, making histone modifications a persistent focus of research for their biological processes and functional significance. Dysregulation of histone modifications can alter the balance between transcriptional activation and repression, thereby contributing to disease onset and progression [1]. Consequently, histone modifications represent multifunctional markers closely associated with disease development, and their exploration in disease pathogenesis has garnered increasing attention.

Histones can be modified in multiple ways, including acetylation, methylation, phosphorylation, ubiquitination, and SUMOylation, modifications long recognised [2,3,4]. With the advancement of high-sensitivity mass spectrometry, various histone acylation marks derived from cellular metabolites have been discovered, such as propionylation, butyrylation, succinylation, and malonylation [5,6,7,8]. Lactylation is a PTM originating from Lactate, occurring on lysine residues of human histones [9]. First identified in 2019 and termed lactylation, it functions as an epigenetic modification regulating and modulating gene transcription [10,11]. Interestingly, Lactate is produced via glycolysis under hypoxic conditions or during bacterial challenges, and lactylation modifications play a significant role in epigenetic regulation within the pathophysiology of multiple diseases [12,13,14]. Given that the mechanisms of histone lactylation in ocular diseases are currently poorly understood [15], further investigation into its pathological relevance is highly warranted.

Lactylation modification participates in regulating tumourigenesis and metabolic reprogramming. In ocular research, novel evidence demonstrates that lactylation is closely associated with the initiation and progression of ocular diseases, including myopia, ocular malignant melanoma, retinopathy, uveitis, and macular degeneration. At the cellular level, retinal capillary endothelial cells, retinal pigment epithelial (RPE) cells, Müller cells, retinal ganglion cells (RGC), and photoreceptor cells participate in lactate metabolism. While the role of histone lactylation has been extensively studied in various cancers, its involvement in ocular diseases remains an emerging frontier. This review/study aims to explore the potential links between lactate metabolism and epigenetic modifications in the eye, providing a framework for future mechanistic studies.

## 2. Lactate Metabolism and the Eye

**Figure 1:**
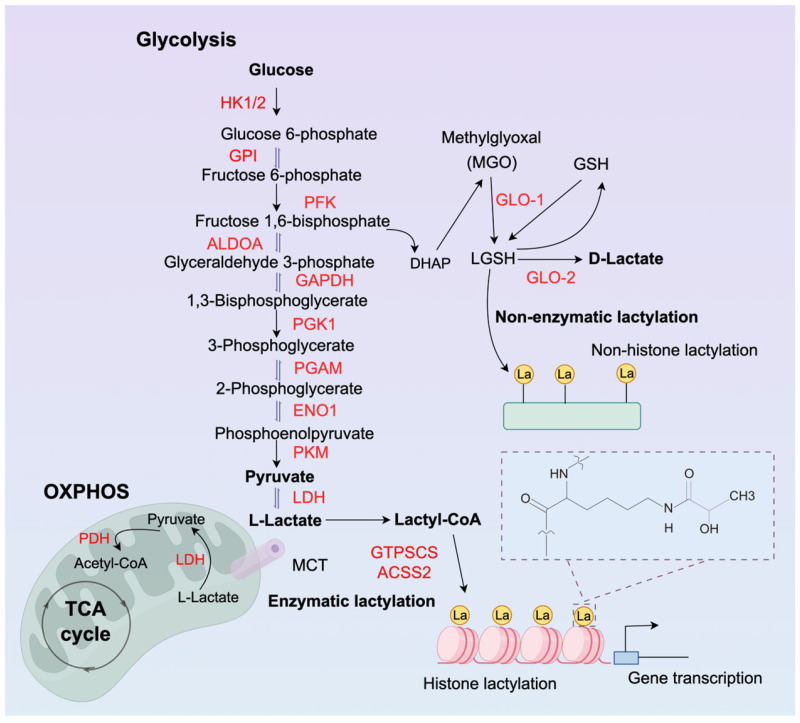
The normal glycolytic pathway produces lactate for lactylation and a simplified oxidative phosphorylation process. Overview of lactylation. Substrates for lactylation include histones and non-histones; types of lactylation encompass enzymatic and non-enzymatic pathways.

Normal cells metabolise glucose via glycolysis and mitochondrial oxidative phosphorylation (Figure 1). Under aerobic conditions, pyruvate derived from glycolysis is channeled into the mitochondria to fuel oxidative phosphorylation (OXPHOS), a process that consumes oxygen and generates water and ATP. Under anaerobic conditions, glucose can be broken down via glycolysis into two molecules of pyruvate, which is then reduced to lactate by lactate dehydrogenase (LDH). Lactate, initially considered a metabolic waste product, is now recognised as an important component, primarily cleared through OXPHOS. Notably, many tumour cells, such as ocular melanomas, rely on lactate for energy production, converting glucose into lactate even under aerobic conditions—a phenomenon termed the Warburg effect [16,17].

### 2.1. The Unique Metabolic Paradox of the Eye: Balancing Optical Clarity and Energetic Demand

Unlike most peripheral tissues, the eye operates under a unique physiological paradox: it must maintain strict optical transparency to transmit light while simultaneously meeting some of the highest metabolic demands in the human body [18]. This functional constraint dictates a highly specialized, “lactate-centric” metabolic landscape. Rather than a mere metabolic waste product of ischemic stress, lactate is produced continuously at high baseline levels as a fundamental requirement for vision.

The posterior segment faces a different challenge. Retinal photoreceptors possess an exceptionally rapid energy turnover required for phototransduction and the daily renewal of outer segments [19]. Paradoxically, despite being adjacent to the oxygen-rich choroidal blood supply, photoreceptors predominantly utilize aerobic glycolysis—the well-known Warburg effect. This metabolic reprogramming allows for the extremely rapid generation of ATP and anabolic intermediates needed for cell maintenance, concurrently producing copious amounts of lactate even in the presence of oxygen.

The retina exhibits high metabolic activity, demonstrating significant energy requirements (Table 1). It primarily utilises aerobic glycolysis (Warburg effect) to convert most glucose into lactate [20,21,22,23,24]. Compared to the well-vascularized inner retina, the outer retina relies more heavily on glycolysis; roughly 80% of its glucose supply is diverted toward lactate production, in contrast to only 20% in the inner retina [18,19]. In the murine retina, lactate production has been shown to consistently exceed that of pyruvate. Across various mammalian species, intraretinal lactate concentrations range from 5–50 mmol/L, a level strikingly higher than the 1–2 mmol/L typically observed in human peripheral blood [25,26]. Illumination induces a reduction in oxygen and glucose demand within the outer retina relative to darkness. Conversely, the inner retina maintains a consistent metabolic rate, with activity under constant illumination mirroring that observed in the dark [25]. The outer retina relies primarily on photoreceptor cells and the RPE, while the inner retina also requires energy turnover, particularly for sustained energy supply to maintain membrane potential during neural transmission between bipolar cells and RGCs [27,28]. Although once considered a metabolic waste product, lactate is now recognized for its critical roles in neuroprotection. Specifically, Müller cells support the survival of RGCs by serving as metabolic reservoirs and maintaining glutamate homeostasis within the synaptic cleft [29,30,31,32].

To preserve a clear optical axis, anterior structures such as the cornea and the lens must remain entirely avascular. The absence of a direct blood supply, combined with the evolutionary minimization of light-scattering mitochondria, forces these tissues into a chronic state of hypoxia which maintains an oxygen tension of ~1% in the lens core. Consequently, these tissues are compelled to rely heavily on anaerobic glycolysis. The cornea, for instance, converts a vast majority of its consumed glucose into lactate [33,34]. This glycolytic flux is not a sign of metabolic inefficiency, but a necessary adaptation to generate ATP while keeping the tissue dehydrated and transparent.

The cornea requires energy metabolism to maintain its transparency (Table 1). Approximately 85% of the glucose absorbed by the cornea is metabolised into lactate, indicating a high dependence on glycolysis [33,34]. Reflecting their lower mitochondrial content relative to endothelial cells, the epithelium and stroma generate the majority of lactate. This lactate does not diffuse forward; instead, it must pass entirely through the endothelial layer, providing the necessary substrate to fuel its metabolic pumps [35]. Removal of the epithelium reduces corneal lactate production by 50% [36]. Regulation of glycolysis and lactate generation is primarily mediated by phosphofructokinase (PFK), which increases with rising pH. However, not all ocular cells require aerobic glycolysis (Table 1). The lens epithelium receives only 1% oxygen, placing lens cells under hypoxic conditions. This is crucial for maintaining lens transparency and preventing nuclear cataracts [37,38,39,40]. Glycolysis not only provides energy metabolism in human lens epithelial cells but also helps them persistently resist apoptosis induced by endoplasmic reticulum stress and reactive oxygen species.

In summary, it is evident that the role of lactate in maintaining corneal and lens transparency is inconsistent. The cornea, possessing an endothelial pump that facilitates material exchange, generates substantial lactate. Conversely, the lens lacks such a pump, resulting in significantly reduced lactate production from glycolysis. We have also outlined clinical applications of ocular lactate metabolism, including potential pathologies arising from metabolic abnormalities and therapeutic targets (Table 1). It may provide valuable insights for the treatment of other eye diseases.

Together, these evolutionary adaptations—hypoxic glycolysis in the anterior segment and aerobic glycolysis in the posterior segment—render the eye a naturally “lactate-abundant” organ. However, the accumulation of acid would quickly disrupt tissue homeostasis. Therefore, this unique metabolic design necessitates a highly structured system for lactate compartmentalization and transport.

### 2.2. The Lactate Shuttle Mechanism in the Eye

Lactate serves as a vital metabolic substrate for various cell types within the eye, many of which lack the endogenous capacity for its synthesis and thus depend on exogenous supply. This necessitates a bridge to mediate its transport, a process termed the lactate shuttle mechanism. Lactate shuttle flux is regulated by Lactate concentration gradients, pH gradients, and redox states. Lactate traverses the cell membrane via lactate transporters (MCTs), a class of proton-coupled transporters. The MCTs primarily involved in transporting Lactate produced within the eye are MCT1 and MCT4 [41,42,43,44].

Given the obligate high glycolytic flux required for ocular function, the uncontrolled intracellular accumulation of lactate would inevitably lead to lethal acidification and osmotic stress. To prevent this, the eye employs a strict spatial division of labor, compartmentalizing lactate production and lactate clearance or utilization. This dynamic compartmentalization is mechanically bridged by the Lactate Shuttle, heavily reliant on proton-coupled monocarboxylate transporters, primarily MCT1 and MCT4, acting as precise directional valves [36].

In the anterior segment, particularly the cornea, lactate compartmentalization manifests as a unidirectional clearance system. The highly proliferative corneal epithelium generates substantial amounts of lactate via aerobic glycolysis [36]. To maintain corneal deturgescence and prevent edema, this lactate cannot remain stagnant. It is sequentially shuttled through the stroma and actively extruded by the corneal endothelium into the aqueous humor. This precise spatial regulation is mediated by the asymmetrical distribution of MCT1, MCT2, and MCT4 across the endothelial barrier; disruption of this specific transporter function directly culminates in pathological lactate accumulation and corneal edema.

Beyond simple metabolite excretion, the ‘lactate shuttle’ mediated by MCTs establishes a sophisticated metabolic coupling between distinct ocular cell layers [45]. For instance, the directional transport of lactate from photoreceptors to Müller glia or RPE cells—facilitated by the spatial partitioning of MCT1 and MCT4—is not merely a waste-clearance mechanism but a vital signaling flux. This intercellular flux directly elevates local L-lactyl-CoA pools in recipient cells, thereby serving as a prerequisite for histone and non-histone lactylation. Such compartmentalized metabolic-epigenetic crosstalk is central to maintaining ocular homeostasis and driving disease progression under hypoxic or hyperglycemic stress.

The mechanism governing retinal lactate shuttling is considerably more complex, with numerous differing perspectives emerging. A metabolic ecosystem exists between Müller cells, RPE cells, and photoreceptor cells, wherein lactate produced by one cell type may be utilised by others [45,46,47]. Most research has focused on the outer retina, yet the dynamics and extent of this shuttle within the inner retina remain contentious. It was only when studies demonstrated that inhibiting MCTs and their auxiliary proteins led to reduced ERG b-wave amplitude and oscillatory potentials—the b-wave representing bipolar cell signals—that Müller cell-derived lactate was proven to be transported to the inner retina. The precise mechanism was fully elucidated in a mouse study where genetic deletion of *PKM2* and *LDH-A* reduced both ERG a-waves and b-waves [48,49,50,51]. Although the specific transporter protein remains unidentified, FRET-based genetic nanosensors revealed that MCT2 facilitates the transport of Müller-generated lactate to inner bipolar cells for functional action.

The posterior segment utilizes compartmentalization to establish a highly complex metabolic ecosystem (symbiosis). In the retina, spatial segregation occurs between distinct, closely apposed cell layers [46,47]. Photoreceptor cells act as the primary lactate “source,” rapidly extruding massive amounts of lactate via MCT4. Instead of mere waste clearance, adjacent RPE and Müller glia act as the metabolic “sink.” They actively uptake this extracellular lactate via MCT1 to fuel their own mitochondrial oxidative phosphorylation. This elegant compartmentalization ensures that circulating glucose is spared specifically for the high-demand photoreceptors, while supporting the energetic needs of the surrounding support cells.

The partnership between RGCs and Müller cells represents another major discovery in the retinal lactate shuttle mechanism. Subsequently, RGCs can take up Müller-generated lactate for oxidative phosphorylation and ATP production. Initially thought to be unidirectional, subsequent research suggests lactate shuttling between RGCs and Müller cells may be bidirectional, with both cell types capable of recycling their own lactate and potentially protecting the optic nerve [23,24,46,52,53]. However, conclusive evidence remains lacking, necessitating further investigation to confirm this hypothesis.

In summary, the ocular metabolic landscape is governed by a delicate equilibrium between optical transparency and high energetic flux, culminating in a distinctive physiological lactate-rich microenvironment. This sophisticated compartmentalization and the regulated shuttling of lactate via MCTs extend beyond mere metabolic waste management; they establish a unique biochemical milieu. Such high-density, localized lactate concentrations serve as the requisite substrate for lysine lactylation (Kla), forging a direct mechanistic link—a metabolic-epigenetic axis—that translates metabolic shifts into sustained gene reprogramming. Deciphering this metabolic foundation is, therefore, a prerequisite for understanding how lactylation orchestrates the pathogenesis and progression of ocular diseases.

## 3. Lactylation Modification in the Eyeball

### 3.1. Mechanism of Protein Lactylation

Cellular metabolites and products, such as acetyl-CoA, succinyl-CoA and acyl-CoA, can be recruited to chromatin to participate in regulatory functions. Within epigenetic modifications, these metabolites can influence metabolic processes, subsequently affecting gene expression [9,54,55,56]. Short-chain acylation of lysine residues in eukaryotic proteins represents novel post-translational modifications, encompassing acetylation, butyrylation, propionylation, and isobutyrylation [57]. L-Lactate, the predominant form of Lactate, may participate in post-translational modification regulation.

In 2019, Zhang et al. first identified Kla as a novel histone post-translational modification (HPTM) and elucidated its role in metabolic regulation [10]. Using high-resolution liquid chromatography-tandem mass spectrometry (HPLC-MS/MS), they identified a characteristic mass shift of 72.021 Da on lysine residues, corresponding to the addition of a lactyl group. Their initial screening identified 26 and 16 histone Kla sites in human MCF-7 cells and mouse bone marrow-derived macrophages (BMDMs), respectively. Building on this discovery, Zhang et al. demonstrated that histone Kla levels are directly modulated by lactate concentration; specifically, stimulating cells with varying glucose concentrations induced both lactate production and histone Kla in a dose-dependent manner. This established a critical link between glycolytic flux and epigenetic programming.

Does the source of lactate influence lactylation? Their research indicates that both endogenous and exogenous lactate stimulate Kla, primarily dependent on lactate concentration. Having established the aforementioned upstream mechanism, they proceeded to explore the more intricate downstream mechanisms. Following treatment of BMDMs, M1 macrophage polarisation increased lactylation levels within 24 h, primarily acting on H3K18la. Histone Kala was reduced using LDHA inhibitors [10]. Their research focused on the role of the classical metabolic enzyme ARG1 protein in macrophage polarisation. Elevated ARG1 levels synchronised with Kla levels, appearing 12 h after nitric oxide synthase (NOS) expression.

**Figure 2:**
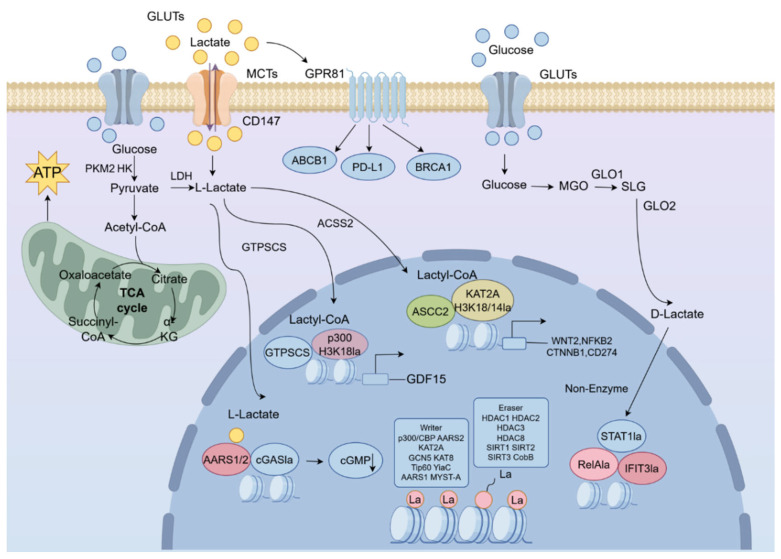
Mechanisms of Protein Lactylation Regulation and Signal Transduction. Mechanisms and regulation of lactylation. Lactate shuttles between cells via MCTs and enters cells as an energy substrate. It also acts as a signalling molecule by binding to the cell surface receptor GPR81, mediating cellular communication. Once activated as lactyl-CoA, lactate functions as a lactyl-donor. AARS (AARS1) functions as a lactylation writer, while HDACs and sirtuins are known erasers. Image created using FigDraw (www.figdraw.com, accessed on 1 March 2026).

Non-histone lactylation modifications have also been functionally validated [58]. Research elucidated a spontaneous, non-enzymatic lactylation mechanism mediated by S-lactoylglutathione (LGSH). Distinct from canonical glycolytic intermediates, LGSH is a key component of the glyoxalase system; it is synthesized from methylglyoxal and glutathione and subsequently serves as a donor by transferring its lactyl moiety to protein lysine residues [59]. Recent study elucidated a non-Lactate-derived molecular mechanism of lactylation [60]. Downregulation of the glycolytic enzyme GLO2 within immune cells led to accumulation of the metabolic molecule SLG (Figure 2), triggering D-lactylation modifications on cytoplasmic proteins—including immune recognition and regulatory molecules—thereby negatively regulating immune responses and inflammatory reactions [60]. Non-enzymatic D-lactylation occurs spontaneously without the requirement for enzymatic catalysis or ATP. This modification is primarily mediated by LGSH, a key intermediate of the glyoxalase system derived from the highly reactive glycolytic byproduct methylglyoxal (MGO). LGSH can directly transfer its D-lactyl group to protein lysines through a proximity-driven chemical reaction [60]. While L-lactylation is associated with physiological signaling, elevated D-lactylation often reflects glycation stress and is closely linked to the accumulation of advanced glycation end products (AGEs), particularly in metabolic disorders like diabetic retinopathy where MGO levels are significantly elevated.

Lactate, the terminal product of glycolysis, serves as a primary source of lactyl groups upon intracellular accumulation. Elevated lactate levels—characteristic of hypoxia, the Warburg effect in malignancies, or inflammatory states—establish the requisite substrate pool for lactylation. However, this process is strictly conditional, depending on the enzymatic conversion of lactate into lactyl-CoA. Lactate must be ligated with coenzyme A by specific synthetases to form lactyl-CoA, which then functions as the proximal donor for protein lactylation reactions. For instance, the utilization of L-lactate for lactyl-CoA generation is regulated by signaling cues: activation of the epidermal growth factor receptor (EGFR) triggers ERK-mediated phosphorylation of acyl-CoA synthetase 2 (ACSS2) at Ser267. This modification promotes the formation of a complex between ACSS2 and lysyl acetyltransferase 2A (KAT2A) [61]. Within this complex, ACSS2 serves as a lactyl-CoA synthase to convert lactate into lactyl-CoA, while KAT2A acts as the lactyltransferase that facilitates tumor immune evasion (Figure 2). The enzymatic execution of Kla is not directly driven by lactate itself but necessitates a high-energy substrate activation. Within the lactate-rich ocular environment, L-lactate is first catalyzed into L-lactyl-CoA by ACSS2, a process strictly dependent on ATP and coenzyme A (CoA-SH). As the central lactyl donor, L-lactyl-CoA enables p300 or AARS1 to transfer the lactyl moiety onto lysine residues of histones or non-histone proteins [54]. This biochemical cascade—bridging metabolic flux to epigenetic signaling via the ‘Lactate-Lactyl-CoA-Transferase’ axis—constitutes the molecular foundation for how metabolic shifts in ocular tissues dictate gene expression and cellular dysfunction. Zhao et al. subsequently identified the lactyl-CoA synthase, guanosine triphosphate (GTP)-specific SCS (GTPSCS) (Figure 2). This protein forms a complex with p300, promoting the production of lactyl-CoA and H3K18la, and elucidates its function within glial cells [62].

The enzymatic activation of L-lactate into L-lactyl-CoA is primarily mediated by ACSS2 [54]. In this ATP-dependent process, L-lactate serves as a substrate, where the carboxyl group of lactate is activated by ATP to form a lactyl-AMP intermediate, subsequently reacting with coenzyme A (CoA-SH) to generate L-lactyl-CoA. This metabolic activation is critical as it provides the high-energy thioester bond required for subsequent lysine lactylation. The regulation of enzymatic lactylation is intricately linked to intracellular metabolic homeostasis. The catalytic activity of ACSS2 and p300 is sensitive to the L-lactate/pyruvate ratio and the availability of ATP and CoA-SH. Under conditions of high glycolytic flux (hypoxia or the Warburg effect), the accumulation of lactate promotes the nuclear translocation of ACSS2, thereby increasing the local concentration of L-lactyl-CoA and driving site-specific histone lactylation to modulate gene expression.

### 3.2. Regulation of Protein Lactylation

N-lacetylation modifications can be regulated through enzymatic or non-enzymatic pathways, with enzymatic regulation being the most prevalent. Under enzymatic control, three distinct enzymes perform specific functions, acting as the “writer”, “eraser”, and “reader” (Figure 2). The “writers” and “erasers” respectively add or remove lactyl groups onto amino acid residues such as lysine and glycine. The “readers” convey the critical epigenetic information carried by these modifications.

Following its synthesis, L-lactyl-CoA is utilized by specific lactylation transferases (writers) to modify lysine residues [58]. P300, a well-known histone acetyltransferase (HAT), has been identified as a major enzymatic writer that facilitates the transfer of the lactyl group from L-lactyl-CoA to histones (H3K18). P300 possesses a unique acyl pocket capable of binding diverse acyl groups, rendering it a crucial writer for numerous protein acylations [58]. Beyond histones, p300 and its homologue CBP (cAMP response element-binding protein) function as non-histone acylation writers. PKM2-driven lactate excess induces association between Twist1 and p300/CBP, leading to Twist1 lysine 150 (K150la) lactylation. KAT5/TIP60 acts as an acyltransferase writer, with Vps34 lactylation (at lysine-356 and lysine-781) enhancing its association with Beclin1, Atg14L, and UVRAG [63]. This entire process is activated by ULK1 directly interacting with LDHA.

Distinct from the canonical Coenzyme A (CoA)-dependent pathway utilized by p300, AARS1 functions as a non-canonical lactyltransferase via an ATP-dependent mechanism. It activates L-lactate to form a lactyl-AMP (adenylate) intermediate, allowing it to directly catalogue protein lactylation in vivo and in vitro [64,65]. Specifically, AARS1 targets YAP at Lys90 and TEAD1 at Lys108. Conversely, within the acyl-CoA-dependent framework, KAT8 serves as the writer for eEF1A2 lactylation [66]. This modification enhances eEF1A2’s translational efficiency, thereby promoting the synthesis of multiple oncogenic proteins.

Another crucial component in lactylation modification is the eraser, with the earliest identified being HDACs (HDAC1-3) and SIRTs (SIRT1-3), primarily acting upon L-lactylation [6,10,67,68]. HDAC1-3 can also exert its de-lactylating activity on D-lactylation. The lactylation of METTL16 at position K229 is inhibited by SIRT2, thereby exerting its de-lactylation activity [69]. YiaC and CobB were identified in Escherichia coli MG1655 as lactyl transferase and de-lactylase, respectively; the former catalyses the addition of Kla, while the latter removes this post-translational modification [70].

## 4. Lactylation Modification and Ocular Diseases

In recent years, lactate modification has been implicated in disease onset and progression through its role in various physiological processes. Significant advances have been made in understanding the molecular mechanisms of lactate modification signalling pathways and their contribution to disease development. Research indicates that lactate may play a role in glaucoma, myopia control, uveitis, retinal disorders, age-related macular degeneration, and ocular tumours.

### 4.1. Glaucoma

**Figure 3:**
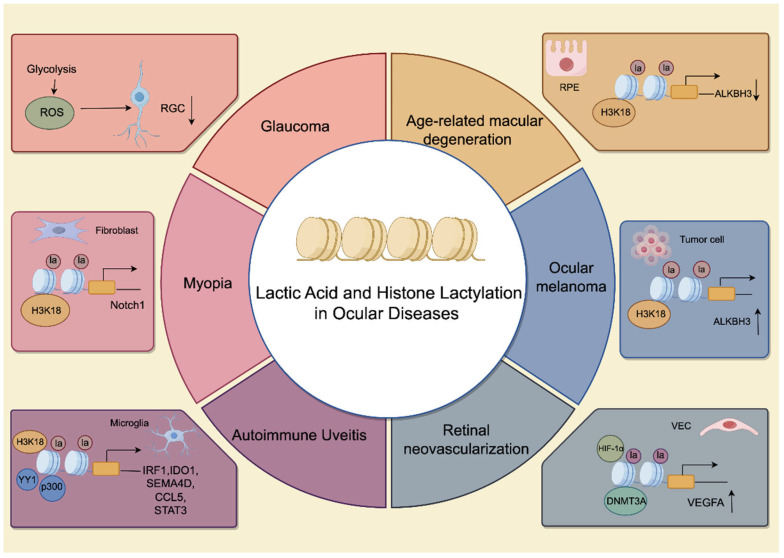
Diverse roles of histone lactylation in the pathogenesis of ocular diseases. The diagram illustrates distinct molecular pathways involving Lactate and histone H3 lysine 18 lactylation (H3K18la) across different ocular pathologies. Specific cell types (RGCs, RPE, VECs, Microglia, and Fibroblasts) exhibit unique gene regulatory networks driven by lactylation or glycolysis-related metabolic shifts. Key downstream targets include Notch1 (Myopia), inflammatory mediators (Uveitis), VEGFA (Neovascularization), and ALKBH3 (AMD and Melanoma), highlighting the dual role of lactylation in promoting or inhibiting disease progression depending on the cellular context. Image created using FigDraw (www.figdraw.com, accessed on 1 March 2026). Abbreviations: RGC, retinal ganglion cell; RPE, retinal pigment epithelium; VEC, vascular endothelial cell.

As a chronic optic neuropathy, glaucoma is defined by the progressive degeneration of RGCs [71]. The disease process typically initiates with the gradual loss of RGC axons, ultimately culminating in the loss of their somata and permanent visual impairment. Intraocular pressure (IOP), advanced age, familial predisposition, and non-Caucasian ethnicity constitute significant risk factors for its onset [72]. Increased IOP impairs ocular perfusion and nutrient transport, further intensifying the metabolic burden on retinal ganglion cells [73]. Energy supply to RGCs is derived from Müller cells, including lactate (Figure 3). Once mitochondrial dysfunction occurs, it triggers glucose metabolism disorders, compromising the neurotrophic conditions essential for RGC maintenance and survival. Disturbances in lactate levels may induce metabolic acidosis and trigger programmed cell death [74].

Evidence from experimental models suggests that L-lactate promotes RGC survival and preserves visual function through lactate metabolism and ATP production, highlighting its potential as a neuroprotective strategy for glaucoma [46]. For instance, overexpression of the lactate transporter MCT2 has been shown to protect RGCs and improve energy homeostasis in glaucoma-related models [75]. However, it is important to note that these protective roles have been primarily characterized in preclinical settings, and direct evidence of protein lactylation within glaucomatous tissues remains insufficient. Whether lactate-mediated modifications, rather than just its metabolic utilization, represent a primary mechanism for RGC protection in clinical glaucoma remains an open question. Bridging this gap by investigating the direct presence and regulatory role of lactylation in the glaucomatous retina represents a crucial future research direction.

### 4.2. Myopia

As a pressing global health concern, myopia represents a rapidly expanding area of biomedical investigation [76]. Increasing evidence indicates that myopia arises from multiple factors including environmental exposure and genetics [77]. From a pathomechanistic perspective, choroidal blood perfusion (ChBP) and scleral hypoxia drive myopia progression. Fibroblast-to-myofibroblast transformation (FMT) in scleral fibroblasts promotes extracellular matrix (ECM) remodelling, manifested by elevated expression of glycolytic key enzymes and increased lactate levels. Inhibiting glycolysis or lactate production reverses FMT and halts myopia progression. Evidence suggests that hypoxia induced by intensive near work and hyperinsulinemia from high-sugar diets both trigger scleral glycolysis, potentially serving as convergent pathways in the pathogenesis of myopia. The resulting accumulation of glycolytic lactate promotes the FMT and myopic progression via H3K18 lactylation and the concomitant activation of Notch1 signaling (Table 2). These findings elucidate the mechanism of scleral ECM remodeling and underscore the regulatory role of protein lactylation in hypoxia-mediated myopia [78]. These findings provide a theoretical framework for novel myopia prevention strategies. While further clinical validation is required, it is hypothesized that managing the timing of intensive near-work—particularly by avoiding it during the postprandial insulin peak following high-carbohydrate meals—could potentially mitigate the synergistic pro-myopic effects of metabolic and hypoxic stressors.

Retinal metabolic shifts and abnormal signaling precede structural scleral remodeling. In contrast to the histone-specific lactylation that drives scleral remodeling, retinal lactylation exhibits a distinct pattern involving a broader array of proteins. In the retina of form-deprivation myopia (FDM) models, 124 Kla sites across 92 proteins were upregulated, while 3 sites across 3 proteins were downregulated, with the splicing factor SUGP2 showing five significantly altered sites [79]. These modifications are heavily enriched in pathways such as glutathione metabolism, glycolysis, and the HIF-1 signaling pathway. Unlike the scleral mechanism—which focuses on H3K18 lactylation-mediated gene expression for ECM remodeling—the retina appears to utilize lactate-driven modifications on non-histone proteins to modulate early-stage metabolic reprogramming and signal initiation in myopia progression.

### 4.3. Uveitis

Uveitis is a relatively complex ocular immunological disorder primarily affecting the uvea, the middle layer of the eyeball. Etiologically, uveitis is categorised as infectious (antigen-mediated), non-infectious (immune-mediated), or pseudo-inflammatory (cancer-associated). Non-infectious uveitis constitutes one such category, primarily manifesting as symptoms of autoimmune or systemic inflammatory diseases, including juvenile arthritis, Vogt–Koyanagi–Harada disease, sarcoidosis, and Blau syndrome [80,81,82]. Both innate and adaptive immune systems participate in the inflammatory pathways of uveitis. The former predominantly functions in infectious uveitis, while the latter primarily operates within the adaptive immune system.

Upon pathogenic stimulation, resident microglia are the first responders that undergo activation. Subsequently, they facilitate the recruitment of infiltrating macrophages from the peripheral blood. Together, these myeloid cells act as antigen-presenting cells to restimulate Th17 cells, thereby perpetuating the adaptive autoimmune cascade. The pathogenesis of autoimmune uveitis primarily involves disruption of the blood-retinal barrier and activation of Th17 cells and microglia [83]. Lactylation functions as a critical regulatory mechanism governing Th17 polarization. Dysregulation of initial CD4+ T cell differentiation, influenced by metabolic reprogramming from oxidative phosphorylation to glycolysis and lactate metabolism, is associated with autoimmune uveitis [84]. Research indicates that the lactylation process regulates CD4+ T cell differentiation, with Ikzf1 lactylation at the Lys164 site modulating Th17-associated genes such as *Runx1*, *Tlr4*, *IL-2,* and *IL-4* (Table 2). However, the experimental autoimmune uveitis (EAU) model was established in immunized mice, with Th1 and Th17 cells isolated from the spleen and inguinal lymph nodes rather than from ocular immune cells [83]. In EAU, increased YY1 lactylation occurs in retinal microglia. Conversely, YY1 lactylation enhances microglial activation, and inhibiting this process attenuates EAU progression [85]. Furthermore, YY1 lactylation induces a cascade of inflammatory genes [85], including downstream inflammatory mediators such as *STAT3*, *CCL5*, *IRF1*, *IDO1*, and *SEMA4D* (Table 2). This further demonstrates that lactate-induced lactylation modifications participate in the pathogenesis of autoimmune uveitis.

**Table 2:** Lactate-Modified Genes and Signalling Pathways in Ocular Diseases.

Disease	Lactylation-Modified Genes and Sites	Molecular Target/Signaling Pathway	Clinical Significance or Potential Treatment
Myopia	*H3K18la*	*Notch1*	Alleviate FMT and the development of myopia [78]
Autoimmune uveitis	*Ikzf1* (*Lys164*)	*IL-2*, *IL-4*, *Runx1*, *TIr4*	Promote Th17 cell differentiation, induce uveitis [83]
	*YY1*, *P300*	*STAT3*, *CCL5*, *IRF1*, *IDO1*, *SEMA4D*	*YY1* induces microglial uveitis [85]
Age-related macular degeneration	*ALKBH3* (*H3K18*)	*ALKBH3*/*VEGFA*	RPE degeneration to CNV [84]
Retinal disease	*YY1* (*K183*), *P300*	*P300*/*YY1*/*FGF2*	Targeted proliferative retinopathy [86]
	*FTO* (*H3K18*)	*FTO*/*CDK2*/*YTHDF2*	Targeted DR [87]
	*DNMT3A*, *HIF-1α*	*DNMT3A*/*HIF-1α*/*VEGFA*	Angiogenic therapy [15]
	*SEMA6A* (*H3K9*, *H3K18*), *P300*	*PRMT5*, *RHOA*	Ischemic retinopathy pathological angiogenesis [88]
	*POSTN* (*H4K8*)	*HIF-1α*	Targeted angiogenic retinopathy [89]
Ocular melanoma	*H3K18*	*ALKBH3*, *SP100A*	Targeted melanoma therapy [90]
	*LSD1* (*H3K4*)	*LSD1*/*FosL1*/*TRIM21*	Melanoma resistance [91]
	*H3K18*	*YTHDF2*/*PER1*/*TP53*	Accelerate the progression of melanoma [92]

### 4.4. Age-Related Macular Degeneration

Age-related macular degeneration (AMD) is a complex ocular disorder arising from genetic factors, lipid metabolism, oxidative stress, and ageing, with its primary clinical manifestations being loss of central vision and blindness [93,94]. Characterised by severe degeneration of photoreceptor function in the macular region due to damage to RPE cells, AMD presents in two advanced forms: dry AMD and neovascular AMD. Dry AMD denotes vision deterioration stemming from RPE degeneration and the neurons it supports [94,95]. Approximately 10% of dry AMD patients progress to neovascular AMD. Neovascular AMD manifests as choroidal neovascularisation (CNV), causing severe visual impairment. Preventing the progression from dry to neovascular AMD represents a crucial strategy for averting vision loss.

Current research has primarily focused on the lactylation modification of neovascular AMD. Research indicates that the m1A demethylase *ALKBH3* removes methylation from HK2, thereby activating glycolysis in the RPE (Table 2). By analyzing single-cell RNA-Seq (scRNA-seq) data from two patients with neovascular AMD and two control subjects, this study revealed significant differential expression of *ALKBH3* in RPE cells. However, histone deacetylation levels were not directly measured. This is potentially implicated in increased lactate production, which subsequently promotes histone lactylation at H3K18 and *ALKBH3* transcription [84]. This proposes a potential link between a positive feedback loop, ultimately triggering RPE degeneration. Separately, metabolomic analysis of serum from neovascular AMD patients revealed lactate’s role in promoting angiogenesis and M2-like macrophage aggregation, demonstrating pyruvate dehydrogenase kinase (PDK) as a key pathogenic factor in AMD [96]. However, the study measured lactate levels rather than directly assessing altered histone lactylation. This further highlights lactylated modifications in AMD patients, though the underlying mechanism remains unexplored. Further research on the lactation of dry AMD remains necessary.

### 4.5. Retinal Diseases

Diabetic retinopathy (DR) is a microvascular disorder of the retina caused by the long-term effects of diabetes. The pathogenesis of DR is associated with the generation of oxidants and oxidative stress within retinal tissues. The secretion of vascular endothelial growth factor (*VEGF*), driven by mitochondrial dysfunction, inflammation, and hypoxia, consequently is potentially implicated in vascular and neuronal apoptosis [89]. *FTO* mediates microvascular leakage in diabetes through endothelium-peripheral cell interactions. Its upregulation is precisely triggered by histone deacetylation processes [87]. By demethylating and regulating *CDK2* mRNA stability, *FTO* modulates the diabetic retinopathy phenotype.

Retinal angiogenesis constitutes a primary pathological mechanism in multiple retinal disorders, including retinopathy of prematurity (ROP) and diabetic retinopathy (DR), affecting over 100 million children and adults globally. Under hypoxia-induced proangiogenic factor stimulation, vessels exhibit uncontrolled proliferation alongside disorganised and immature vascular architecture. Under hypoxic conditions, increased lactylation of microglia accelerates *FGF2* expression, thereby promoting retinal angiogenesis [86]. The specific mechanism involves lactylation of the non-histone transcription factor YY1 at lysine 183 (K183), regulated by p300. A485, as a p300 inhibitor, significantly suppresses vascularisation. Lactate accumulation induces histone lactylation at H3K9 and H3K18 sites in neovascular endothelial cells during the proliferation phase of oxygen-induced retinopathy, with *PRMT5* identified as a downstream target gene. SMEA6A recruits RHOA and histone acetyltransferase p300 via liquid–liquid phase separation (LLPS), promoting p300 phosphorylation and inducing a lactylation cycle (Figure 3) [88]. *DNMT3A* facilitates lactate entry into the nucleus, where it promotes *VEGFA* upregulation via HIF-1α lactylation, stimulating endothelial angiogenesis and offering potential for targeted therapy in retinal neovascular diseases (Figure 3) [15]. POSTN levels are elevated in monocyte exosomes, Postn knockout reduces retinal neovascularisation, and histone H4K8 levels increase under hyperglycaemic conditions. In peripheral blood mononuclear cells isolated from proliferative diabetic retinopathy (PDR) patients, elevated levels of histone H4K8la were observed. Consistent results were also obtained in mouse mononuclear cells [89]. Further validation using additional human clinical samples is still required.

It is crucial to distinguish between the regulatory role of lactate and the cytotoxic effects of MGO. While both are elevated during metabolic reprogramming (the Warburg effect), MGO is a reactive dicarbonyl primarily formed from the non-enzymatic degradation of dihydroxyacetone phosphate (DHAP) and glyceraldehyde-3-phosphate (G3P) [97]. In the retina, excessive MGO accumulation is potentially implicated in the formation of advanced glycation end-products (AGEs), contributing to oxidative stress in RPE cells (in AMD) and pericyte loss (in DR). The glyoxalase system (Glo1/Glo2), which relies on glutathione (GSH), serves as a critical defense mechanism by converting MGO into D-lactate, highlighting the interplay between glycolytic flux, redox balance, and pathological glycation.

While current ocular research has predominantly focused on enzyme-mediated L-lactylation (p300/AARS1-driven Kla), the role of non-enzymatic D-lactylation remains a critical but unexplored frontier. Given the massive accumulation of MGO in the diabetic retina and aging lens, it is highly probable that D-lactylation of structural proteins contributes to the irreversible cross-linking and metabolic dysfunction observed in DR and cataracts. Future lactyl-proteomic studies specifically targeting D-lactate marks are warranted to validate this pathogenic mechanism in the eye.

### 4.6. Ocular Melanoma

Uveal melanoma (UM) is a common primary intraocular malignancy in adults, with epigenetic alterations playing a role in its pathogenesis and metastasis [98]. BAP1 mutation constitutes a key alteration in melanoma, promoting glycolysis in tumour cells and leading to increased lactate production. The interaction between BAP1 and LDHA exerts subsequent effects on lactate generation in melanoma [99]. Lactate (20 mM) significantly inhibits UM cell proliferation and migration, whilst elevated expression of the lactate transporters MCTA and HCAR1 correlates with the spindle cell histological subtype of UM.

Given the distinct anatomical origins of melanomas and the established role of lactylation in cutaneous subtypes, it is critical to determine whether this epigenetic mechanism plays a similarly significant role in Uveal Melanoma (UM). Histone lactylation promotes transcription of *YTHDF2*, a protein that recognises m6A-modified sites on the tumour suppressor genes *PER1* and *TP53*, thereby facilitating their degradation [97]. Analysis of 82 intraocular melanoma tissue samples revealed significantly elevated levels of histone lactylation compared to controls [92]. Histone lactylation levels are upregulated in melanoma. Another study revealed that *ALKBH3* is specifically upregulated in ocular melanoma due to excessive histone lactylation levels, promoting tumour progression through *SP100A* m1A demethylation. While the activation of GPR81 and the metabolic utilization of lactate certainly contribute to tumor progression, emerging evidence suggests that lactylation-dependent epigenetic remodeling may be the key driver of the specific phenotype observed in UM.

## 5. Potential Therapeutic Approaches for Eye Diseases Targeting Lactate Metabolism

### 5.1. Validated Therapeutic Agents Targeting Protein Lactylation

#### 5.1.1. ALKBH3 Inhibitor in Combination with Anti-VEGF Apixaban

**Figure 4:**
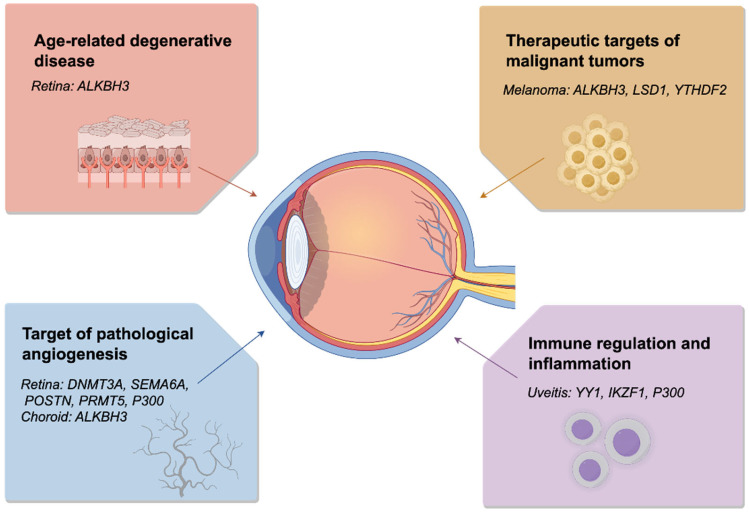
Potential lactylation modification targets in ocular pathologies. Age-related macular degeneration, pathological angiogenesis, malignant tumours, and lactylation targets associated with immunity and inflammation. Image created using FigDraw (www.figdraw.com, accessed on 1 March 2026).

HUHS015, an ALKBH3 inhibitor, is a potent therapeutic. It significantly inhibits DU145 cell proliferation, while ALKBH3 relative activity (RA) decreases with increasing HUHS015 dosage [84]. Research has confirmed that ALKBH3 is involved in the regulation of the expression of the pro-angiogenic factor VEGF-A, thereby promoting the formation of choroidal neovascularisation (CNV). Animal studies demonstrate that the combination of the ALKBH3 inhibitor HUHS015 with the anti-VEGF drug aflibercept produces a synergistic effect, significantly inhibiting the progression of CNV (Figure 4) [84]. This study elucidates the molecular mechanisms of ALKBH3-mediated metabolic reprogramming and epigenetic regulation in the progression of AMD, providing a new theoretical foundation and potential therapeutic targets for the clinical management of this disease.

#### 5.1.2. Metformin

Metformin is a commonly used hypoglycemic drug, typically available as metformin hydrochloride, synthesized from dimethylamine chloride and dicyandiamide. Research has also validated the potential vasoprotective mechanism of the commonly used hypoglycemic agent metformin. Investigators discovered that metformin inhibits glycolytic activity in monocytes, reduces lactate levels, and decreases exosomal POSTN secretion, thereby mitigating retinal neovascularization (Figure 4). This finding provides a molecular explanation for the clinically observed lower risk of retinal neovascularisation in diabetic patients receiving long-term metformin therapy. It also suggests metformin may exert a ‘vascular protective effect’ beyond its hypoglycaemic action by modulating immune metabolism [89]. The study reveals novel mechanisms underlying retinal neovascularization, offering new therapeutic targets for disease management.

### 5.2. Potential Targets for Lactylation-Based Therapy

#### 5.2.1. P300 Inhibitors

CBP/p300 inhibitors are classified into three categories based on their mechanisms of action and target sites within the enzyme structure. The first category comprises catalytic inhibitors that compete with target peptides and acetyl-CoA for the lysine-CoA (Lys-CoA) binding pocket; The second class acts on the acetyl-lysine binding site, thereby preventing or limiting enzyme-chromatin interactions; The third class comprises synthetically derived molecules with distinct chemical structures: NEO1132, NEO2734, and XP-524 [100]. Inhibition mechanism: The p300 inhibitor C646, which inhibits H3K18la writing, blocks hypoxia-induced upregulation of *ALKBH3*/*HK2*/*VEGFA*; conversely, the histone deacetylase inhibitor valproic acid (VPA) promotes their expression (Figure 4) [91]. P300 functions as a dual-activity enzyme, catalyzing both global acetylation and selective lactylation, depending on the intracellular ratio of acetyl-CoA to lactyl-CoA. P300 inhibitors show promise as therapeutic targets for ocular diseases.

#### 5.2.2. DNMT3A Inhibitors

Two classes of DNMT3A allosteric inhibitors are available: structurally related pyrazolones (Compound 1) and pyridazines (Compound 2). Compound 2 exhibits a distinct inhibitory mechanism of action by disrupting DNMT3A protein–protein interactions (PPI) [100]. A study systematically mapped lactate-specific binding proteins for the first time, identifying DNMT3A as a core mediator in lactate nuclear transport and lactylation of HIF-1α within the VEGFA signalling axis. Consequently, it proposes therapeutic strategies targeting the lactate-*DNMT3A*/*HIF-1α*/*VEGFA* pathway, offering novel therapeutic approaches for retinopathy of prematurity (ROP), proliferative diabetic retinopathy (PDR), and neovascular age-related macular degeneration (wAMD), VEGFA signalling axis (Figure 4). This proposes therapeutic strategies targeting the ‘Lactate-*DNMT3A*/*HIF-1α*/*VEGFA*’ pathway, offering novel insights and potential targets for intervening in retinal neovascular diseases such as ROP, PDR, and wAMD [88].

#### 5.2.3. PRMT5 Inhibitors

The first-generation PRMT5 inhibitors are categorized into two types: those targeting the SAM-binding pocket and those targeting the substrate-binding pocket. Novel inhibitors such as covalent PRMT5 inhibitors, targeted degraders, and synthetic lethal inhibitors are classified as second-generation inhibitors. Currently, multiple drugs have entered clinical trials [101]. Research has revealed that SEMA6A activates P300 via liquid–liquid phase separation, driving a histone lactylation-PRMT5 positive feedback loop that persistently fuels pathological angiogenesis. This identifies novel metabolic-epigenetic therapeutic targets for ischaemic retinal disorders [102,103,104,105,106,107]. Numerous PRMT5 inhibitors are currently under development, though most remain in early-stage research. Given PRMT5’s critical role in haematopoiesis, first-generation inhibitors carry risks of side effects including thrombocytopenia, anaemia, and neutropenia.

However, while drugs targeting lactate metabolism hold promise for ophthalmic diseases, certain concerns persist. For instance, the limitation in targeting lactate metabolism for UM is the metabolic parallelism between neoplastic cells and the healthy retina. Physiologically, retinal photoreceptors and Müller glia rely intrinsically on aerobic glycolysis (Warburg effect) for bioenergetics. Thus, inhibiting key glycolytic drivers like LDHA or MCT1/4 carries a high risk of on-target retinal toxicity. To spare the adjacent visual system, future strategies must pivot towards precision delivery methods, such as nanoparticle-mediated transport, rather than systemic metabolic blockade.

## 6. Discussion

The lactylation modification plays a significant role in the onset and progression of ocular diseases, participating in multiple biological processes and pathophysiological mechanisms, thereby providing potential therapeutic targets for eye disease treatment. Targeted therapeutic agents for lactylation have been developed, demonstrating marked efficacy in animal models. Targeting MCT significantly impacts metabolic symbiosis. Disruption of MCT1 function is potentially implicated in lactate accumulation, inhibiting tumour cell growth and glycolysis, as demonstrated by MCT1 inhibitors [67,99]. Evidence suggests that combining MCT-targeted therapies with other treatments yields enhanced therapeutic effects; co-treatment with MYC and MCT1 demonstrates superior outcomes. Further investigation is required to elucidate the mechanisms and therapeutic potential of these agents in ocular diseases, which currently remain unexplored.

Chemotherapy drugs stimulate substantial lactate accumulation, inducing hyper-acetylation modification at lysine 24 of the homologous recombination repair factor BLM helicase. This is potentially implicated in extensive repair of anthracycline-induced DNA double-strand breaks, thereby triggering resistance. The research team identified the marketed anti-tumour drug irinotecan as an agent capable of efficiently inhibiting BLM-K24 lactylation and blocking homologous recombination repair [64,65,108,109]. They further demonstrated the safety and feasibility of irinotecan liposome combined with epirubicin for treating anthracycline-resistant recurrent bladder cancer. This represents the first clinical trial targeting lactylation modifications [65,109,110,111].

Crucially, a distinction must be maintained between established metabolic dysregulation and the specific downstream mechanism of protein lactylation. In many ocular pathologies, such as diabetic retinopathy and age-related macular degeneration, metabolic shifts—including *HIF-1α*-mediated *VEGF* up-regulation, mitochondrial dysfunction, and the Warburg effect—are well-documented. While these processes lead to the accumulation of lactate, they should not be conflated with lactylation itself, which represents a specific epigenetic/post-translational response to such metabolic changes. Currently, direct evidence of histone or non-histone lactylation in ocular tissues remains relatively sparse compared to the vast literature on canonical metabolic pathways. Our discussion of lactylation-driven mechanisms in these diseases is, in part, an extrapolation from findings in systemic immunology and oncology. Therefore, while we propose that lactylation acts as a potential bridge between altered retinal metabolism and sustained gene expression, further high-resolution studies (mass spectrometry-based lactyl-proteomics on human vitreous or donor retinas) are urgently needed to validate these marks as primary drivers in the human eye.

It is essential to situate histone lactylation within the broader and more complex regulatory landscape governing ocular pathologies. Our findings suggest that lactylation does not function as a solitary or dominant driver of processes such as M2 macrophage polarization (ARG1 expression), Th17 differentiation, or angiogenesis. Instead, it should be viewed as a contributing metabolic-epigenetic mechanism that integrates with canonical signaling pathways. Furthermore, the interplay between lactylation and other critical factors—such as mechanotransduction in the extracellular matrix and redox signaling under oxidative stress—likely forms a multi-layered regulatory network. Future studies are needed to delineate the hierarchical relationship between these classical pathways and lactylation to fully understand how metabolic shifts are translated into sustained pathological phenotypes.

A significant hurdle in translating lactylation-targeted therapies to the clinic is the risk of off-target effects on healthy retinal tissue. Unlike many other tissues, the healthy retina—specifically photoreceptor cells—relies heavily on aerobic glycolysis (the Warburg effect) for its energy demands and biosynthetic processes. Therefore, global inhibition of lactate production or histone lactylation might inadvertently impair retinal homeostasis and lead to vision loss. Future research must prioritize the development of targeted delivery platforms, such as ligand-functionalized nanoparticles, to ensure that metabolic modulators are delivered specifically to pathological sites such as subretinal neovascularization or inflamed immune cells while sparing healthy retinal layers.

To maintain scientific rigor, it is necessary to distinguish the varying levels of evidence regarding lactylation’s role in ocular health and disease. Currently, direct clinical evidence in human ocular tissues remains in its infancy, with most findings originating from animal models which provide robust mechanistic insights but may not fully recapitulate human pathophysiology. Furthermore, while in vitro studies using retinal endothelial cells or microglia have elucidated specific molecular pathways, these findings represent simplified biological systems [86]. Significant portions of our current understanding—particularly regarding T-cell exhaustion and macrophage polarization—are hypothetical extrapolations derived from established paradigms in tumor immunology and systemic inflammatory models. While these cross-disciplinary insights provide a valuable roadmap for ophthalmological research, they must be validated through direct proteomic analysis of human ocular samples to move from speculative frameworks to confirmed clinical mechanisms.

In conclusion, while our findings point to a significant correlation between protein lactylation and ocular pathogenesis, these results should be interpreted as a foundation for further research rather than a finalized mechanism. Current research on lactylation remains in its infancy, necessitating systematic analysis of its association with ocular disease development. Future efforts should employ high-throughput sequencing technologies for in-depth exploration, identifying additional lactylation sites, developing novel computational models, and integrating multi-omics approaches to elucidate mechanisms and establish multidimensional target maps. Concurrently, investigations into combination therapies and drug synergies should advance to enhance both safety and efficacy.

## Figures and Tables

**Table 1:** Lactate Production and Functions in Retinal, Corneal, and Lens Cells.

Cell Type	Cellular Subspecies	Lactate Metabolism	Metabolic Abnormality	Clinical Application
Retinal cell	Retinal vascular endothelium	Aerobic glycolysis produces small amounts of lactate and ATP	Rapid and adequate supply of lactate and ATP during neovascularization	Targeting oxidative stress and inhibiting glycolysis serve as strategies for anti-neovascular therapy
	RPE	RPE mitochondria are energized by lactate produced by photoreceptor cells to provide ATP for survival	RPE cells avoid oxidative damage by increasing glycolysis, and large amounts of glycolytic products accumulate and are toxic to photoreceptor cells	RPE mitochondria as a target for retinal degenerative diseases
	Photoreceptor cell	Outer segment-dependent glycolysis produces lactate, which is shuttled to the RPE via MCT1	Under hypoxic conditions, there is a compensatory increase in glycolysis and an increase in lactate production, and under anaerobic conditions, nerve impulses from sensory cells are lost	Conserved oxidative stress targeting as in endothelial cells for vision preservation
	Müller cell	Aerobic glycolysis, gene- rates lactate to protect Müller cells	Müller cells take up glutamate, an excitatory neurotransmitter, and glutamate homeostasis can be toxic to optic nerve cells	Visual protection and blood retinal barrier provide targets
	RGCs	Energy via glycolysis produced by Müller cells	Müller cells take up glutamate, an excitatory neurotransmitter, and glutamate homeostasis can be toxic to optic nerve cells	Delaying Vision Loss in Disease Loss of glutamate homeostasis can be toxic to the optic nerve
Corneal Cell	corneal epithelial cell	Aerobic glycolysis produces large amounts of lactate and ATP	Epithelial removal, 50% reduction in Lactate content	
	corneal stromal cell	Aerobic glycolysis produces large amounts of lactate and ATP		
	corneal endothelial cell	Produces small amounts of Lactate Transportation of epithelial and stromal lactate via MCT1, MCT2, and MCT4	Endothelial cell dysfunction, ca- using decreased lactate efflux.	Increased corneal lactate and corneal edema
Lens cell	LEC	Anaerobic glycolysis pro- duces Lactate		Resistance to apoptosis induced by endo- plasmic reticulum stress and reactive oxygen species

## Data Availability

No new data were created or analyzed in this study. Data sharing is not applicable to this article.
